# Impacts of the Covid-19 Pandemic on Life of Higher Education Students: Global Survey Dataset from the First Wave

**DOI:** 10.1016/j.dib.2021.107659

**Published:** 2021-12-01

**Authors:** Aleksander Aristovnik, Damijana Keržič, Dejan Ravšelj, Nina Tomaževič, Lan Umek

**Affiliations:** Faculty of Public Administration, University of Ljubljana, Slovenia

**Keywords:** Covid-19, University students, E-learning, Academic work, Academic life, Social life, Mental health, Institutions

## Abstract

The Covid-19 pandemic caused by the novel coronavirus SARS-CoV-2 has completely reshaped the lives of people around the world, including higher education students. Beyond serious health consequences for a proportion of those directly affected by the virus, the pandemic holds important implications for the life and work of higher education students, considerably affecting their physical and mental well-being. To capture how students perceived the first wave of the pandemic's impact, one of the most comprehensive and large-scale online surveys across the world was conducted. Carried out between 5 May 2020 and 15 June 2020, the survey came at a time when most countries were experiencing the arduous lockdown restrictions. The online questionnaire was prepared in seven different languages (English, Italian, North Macedonian, Portuguese, Romanian, Spanish, Turkish) and covered various aspects of higher education students’ life, including socio-demographic and academic characteristics, academic life, infrastructure and skills for studying from home, social life, emotional life and life circumstances. Using the convenience sampling method, the online questionnaire was distributed to higher education students aged 18 and over and enrolled in a higher education institution. The final dataset consisted of 31,212 responses from 133 countries and 6 continents. The relationships between selected socio-demographic characteristics and elements of student life were tested by using a chi-squared test. The data may prove useful for researchers studying the pandemic's impacts on various aspects of student life. Policymakers can utilize the data to determine the best solutions as they formulate policy recommendations and strategies to support students during this and any future pandemic.

## Specifications Table

**Table:** 

Subject	Education
Specific subject area	Online learning, academic work, academic life, social life, habits, emotional life, personal circumstances, institutions
Types of data	Excel file, csv file, SPSS file, Table
How the data were acquired	Data were gathered using a web-based survey that was conducted via the open-source web application 1KA (One Click Survey; www.1ka.si) and then converted into .xlsx, .csv and .sav formats.
Data format	Raw, Analysed
Description of data collection	The survey targeted all higher education students, who were recruited by a non-probabilistic sampling technique facilitated by promoting the online questionnaire on various university communication systems around the world as well as on social media.
Data source location	Institution: Faculty of Public Administration, University of Ljubljana City: Ljubljana Country: Slovenia
Data accessibility	Data are presented in this article. Repository name: Mendeley Direct URL to data: http://dx.doi.org/10.17632/88y3nffs82
Related research article	Aristovnik, A., Keržič, D., Ravšelj, D., Tomaževič, N., & Umek, L., Impacts of the COVID-19 pandemic on life of higher education students: A global perspective, Sustainability, 12 (20) (2020) 8438. https://doi.org/10.3390/su12208438

## Value of the Data

• The global dataset for the first wave of the Covid-19 pandemic provides direct and valuable information on the pandemic's impacts on higher education students in various aspects of their lives.
• The dataset provides the most comprehensive data for a sample of 31,212 students across 133 countries and 6 continents (Africa, Asia, Europe, North America, Oceania, South America), allowing geographical comparative examinations.
• The dataset covers various aspects of student life (socio-demographic and academic characteristics, academic life, infrastructure and skills for studying from home, social life, emotional life and life circumstances) and thus provides a rich environment for researchers to examine the interactions of various aspects of student life.
• The dataset involves considerable information for education stakeholders and policymakers to identify the best solutions as they formulate policy recommendations and strategies to support students during this and any future pandemic.
• The dataset could be extended by collecting data during future possible waves of the Covid-19 pandemic using the same questionnaire or a questionnaire adjusted to the post-pandemic era, thereby allowing a longitudinal examination.
• The dataset may be (re)used for further in-depth examinations and as a benchmark for comparing similar data involving the Covid-19 pandemic's implications for student life.

## 1 Data Description

The Covid-19 pandemic caused by the novel coronavirus SARS-CoV-2 has created an unprecedented challenge with drastic consequences for all national systems, including higher education, which had no benchmark or previous experience available [1], [2], [3]. The health crisis supposedly emerged in China during December 2019 and its sudden outbreak saw it begin to spread rapidly across the world. The situation became so serious that the World Health Organization (WHO) declared the Covid-19 outbreak a global pandemic on 11 March 2020 [4,5]. Beyond serious health consequences for a proportion of those directly affected by the virus, the pandemic holds important implications for the life and work of higher education students, considerably affecting their physical and mental well-being [6]. Namely, to curb the spread of Covid-19, educational institutions across the world transferred various courses from onsite to online with a rapid pace [7,8], with online learning (e-learning) thereby becoming a mandatory teaching and learning process of higher education institutions [1]. Many higher education institutions were even encountering e-learning for the first time, making the transition especially demanding for them because almost no time was available to organize and adapt to the new landscape of education [2]. Accordingly, this has created many challenges for both teacher and students.

The global dataset contains raw data on how higher education students perceive the impacts of the first wave of the Covid-19 pandemic on various aspects of their lives on a global level [1]. Data were collected as part of the CovidSocLab [6] project, which served as a working platform for international collaboration and implementation of the Global Student Survey. The project aimed to provide the latest and comprehensive findings in selected research areas in the social sciences. To capture the students’ perceptions on the Covid-19 pandemic's impact, the Faculty of Public Administration at the University of Ljubljana, Slovenia, cooperating with an international consortium of universities, other higher education institutions and students’ associations, launched one of the most comprehensive and large-scale online surveys across the world entitled “Impact of the Covid-19 Pandemic on Life of Higher Education Students” [6].

The online questionnaire targeted higher education students with respect to what life was like for them during the first wave of the Covid-19 pandemic. It comprised 39 mainly closed-ended questions divided into seven sections, namely socio-demographic and academic characteristics, academic life, infrastructure and skills for studying from home, social life, emotional life, life circumstances as well as personal reflections on Covid-19 [1]. At the end, there was an option for the respondents to give their e-mail address in case they would like to be notified about the survey results [9,10].

Certain answer choices (“other” and “not applicable”), open-ended questions (the question on personal reflections on Covid-19) and questions raising anonymity issues (the question on a student's e-mail address) are excluded from the dataset. Hence, it covers a total of 160 items from across various aspects of student life. Detailed information on the measured aspects of student life during the first wave of the Covid-19 pandemic is presented in Table 1. Data are provided in the form of Excel (in .xlsx and .csv formats) and SPSS (in .sav format) files, with rows representing cases and columns designating variables. Specific information for each variable, i.e., name, label, values, remarks, is provided in the codebook (view the Excel file in the .xlsx format or SPSS file in .sav format), while empty cells are considered to be missing values.

**Table 1:** Aspects of higher education students’ life included in the dataset.

Aspect	Number of items
Socio-demographic and academic characteristics	7
Academic Life	1
• Lectures	6
• Tutorials/seminars and practical classes	6
• Supervisions/mentorships	6
• Assessment and workload	6
• Satisfaction with teaching and administrative support	12
• Student performance and expectations	6
Infrastructure and skills for studying from home	17
Social life	18
Emotional life	10
Life circumstances	0*
• General circumstances	10
• Financial circumstances	8
• Support measures and behaviour	47

**Total number of items**	**160**

Note: * introductory statement only.

The final sample consists of 31,212 respondents who shared their perceptions of the first wave of the Covid-19 pandemic's impact. The response rate was 33.1% since 94,246 respondents actually opened the link to the online questionnaire. The participation was unequally distributed throughout the 133 countries from the 6 continents [1]. With 308 responses not providing the information on the country, the participation distribution was the following (see Aristovnik et al. [1]): (1) more than 1000 responses were collected in 10 countries (Poland, Italy, Mexico, Chile, Turkey, India, Ecuador, Bangladesh, Portugal, Slovenia); (2) between 500 and 1000 responses were collected in 7 countries (Romania, Croatia, Pakistan, Indonesia, Brazil, Hungary, Ghana); (3) between 200 and 500 responses were collected in 19 countries; (4) a total of 2911 responses were collected in 41 countries having between 10 and 200 responses; and (5) a total of 130 respondents were collected in 56 countries with fewer than 10 responses; however, these respondents are censored to ensure anonymity. Detailed information on the survey respondents’ main socio-demographic characteristics is presented in Table 2, whereby missing values are excluded from the calculations.

**Table 2:** Socio-demographic characteristics of the survey respondents – mean (SD) or number (%).

Socio-demographic characteristics	Mean/Number (SD/%)
**Age**	
Mean (SD)	23.6 (5.6)

**Gender**	
Male	10,519 (34.6)
Female	19,875 (65.4)

**Status**	
Full-time	26,933 (87.8)
Part-time	3,753 (15.1)

**Level of study**	
First	24,412 (80.1)
Second	4,600 (15.1)
Third	1,460 (4.8)

**Field of study**	
Arts and humanities	3,070 (10.2)
Social sciences	11,147 (37.0)
Applied sciences	9,362 (31.1)
Natural and life sciences	6,517 (21.7)

Note: The final sample consists of 31,212 respondents.

The respondents ranged in age from 18 to 70 years with a mean age of 23.6 years and standard deviation of 5.6 years, with most being female (65.4%). Most respondents were full-time (87.8%) and first-level (80.1%) students. They were mostly studying in the field of the social sciences (37.0%) [1,9].

The content of the dataset can be divided into 10 different aspects of student life (see Aristovnik et al. [1]). Due to the specificity or complexity of items, two aspects (change in habits and personal reflections) were excluded from further consideration. The remaining 8 aspects (from onsite to online lectures, academic work, academic life, social life, emotional life, personal circumstances, role of institutions, measures of institutions) were considered in calculations. Besides some exceptions (view the codebook in the Excel file in the xlsx format or the SPSS file in .sav format), the perceptions (i.e., satisfaction, agreement, importance, or frequency) of individual aspects of student life were primarily assessed using a Likert scale, containing 5 response options with 1 representing the lowest value and 5 the highest value [1,11].

Tables 3 and 4 show the shares of students who selected the top two response options for each item for selected aspects. The shares are presented for the whole sample (general) and by different socio-demographic groups. Moreover, the relationships between selected socio-demographic characteristics and elements of student life were tested using a chi-squared test and considering statistical significance at the 0.05 level. The computed p-values were adjusted using a Bonferroni correction by considering all items covered in the dataset [12].

**Table 3:** The relationships between socio-demographic characteristics (gender and status) and elements of student life.

		Gender			Status		
Aspects/Elements	General	Male	Female	P-value	Full-time	Part-time	P-value
**From onsite to online lectures**							
Satisfaction with video conferences	54.9%	54.0%	56.3%	**<0.001**	56.4%	48.3%	**<0.001**
Satisfaction with recorded videos	43.6%	45.3%	43.4%	0.002	44.6%	40.1%	**<0.001**
Difficult to focus	34.7%	35.6%	34.8%	0.144	35.2%	33.4%	0.035
Adaptation to new learning experience	48.2%	47.8%	49.3%	0.010	49.6%	42.4%	**<0.001**

**Academic work**							
Timely response of teaching staff	46.1%	46.1%	46.8%	0.205	47.2%	41.5%	**<0.001**
Extent of study workload	48.3%	50.9%	47.8%	**<0.001**	49.3%	45.0%	**<0.001**
Satisfaction with support of teaching staff	48.4%	48.3%	49.3%	0.108	49.8%	42.0%	**<0.001**
Satisfaction with support of support staff	43.1%	42.9%	43.9%	0.085	44.3%	38.2%	**<0.001**

**Academic life**							
Access to a computer	16.9%	18.1%	16.5%	<0.001	16.8%	18.3%	0.027
Access to a good Internet connection	41.8%	40.5%	43.1%	**<0.001**	42.9%	36.8%	**<0.001**
Browsing online information	35.6%	33.3%	37.3%	**<0.001**	36.1%	34.4%	0.044
Using online teaching platforms	43.8%	42.1%	45.4%	**<0.001**	44.8%	40.1%	**<0.001**

**Social life**							
Close family member	45.2%	44.9%	45.9%	0.133	45.4%	46.4%	0.241
Someone I live with (e.g., a roommate)	39.1%	42.8%	37.6%	**<0.001**	39.6%	37.6%	0.023
Close friend	51.3%	53.5%	50.8%	**<0.001**	51.3%	53.9%	0.004
Social networks	39.9%	42.4%	39.2%	**<0.001**	40.3%	39.6%	0.435

**Emotional life**							
Bored	56.3%	55.7%	57.5%	0.004	57.0%	55.3%	0.059
Anxious	55.7%	57.8%	55.4%	**<0.001**	56.4%	54.0%	0.006
Hopeful	58.6%	56.2%	60.7%	**<0.001**	59.9%	53.1%	**<0.001**
Frustrated	58.6%	58.6%	59.5%	0.138	59.6%	56.0%	**<0.001**

**Personal circumstances**							
Professional career in the future	51.9%	52.2%	52.6%	0.610	52.9%	48.1%	**<0.001**
Study issues	55.8%	56.5%	56.3%	0.725	56.7%	53.2%	**<0.001**
Personal finances	55.0%	54.4%	56.3%	0.002	56.4%	49.1%	**<0.001**
Future education	55.3%	55.6%	56.0%	0.550	56.3%	52.1%	**<0.001**

**Role of institutions**							
Government	53.8%	53.3%	54.9%	0.011	54.9%	49.7%	**<0.001**
University	53.2%	52.2%	54.5%	<0.001	54.4%	48.4%	**<0.001**
Banks	50.9%	50.7%	51.8%	0.069	51.6%	49.0%	0.003
Hospitals	42.6%	42.3%	43.4%	0.064	43.0%	42.1%	0.289

**Measures of institutions**							
Emergency support for vulnerable population	25.4%	27.4%	24.7%	**<0.001**	25.8%	24.3%	**<0.001**
Childcare for essential workers	28.0%	30.1%	27.3%	**<0.001**	28.3%	27.5%	0.307
Financial assistance for renters	34.2%	35.8%	34.0%	0.001	34.8%	32.9%	0.029
Deferred monthly payments	33.9%	35.2%	33.8%	0.015	34.4%	32.7%	0.039

Note: Bold values denote statistical significance at the 0.05 level (after a Bonferroni p-value correction).

**Table 4:** The relationships between socio-demographic characteristics (level of study and field of study) and elements of student life.

		Level of study				Field of study				
Aspects/Elements	General	First	Second	Third	P-value	Arts and humanities	Social sciences	Applied sciences	Natural and life sciences	P-value
**From onsite to online lectures**										
Satisfaction with video conferences	54.9%	57.9%	49.6%	34.8%	**<0.001**	60.3%	55.7%	58.2%	50.7%	**<0.001**
Satisfaction with recorded videos	43.6%	46.5%	35.9%	28.5%	**<0.001**	46.6%	44.4%	46.6%	39.5%	**<0.001**
Difficult to focus	34.7%	34.6%	38.2%	31.7%	**<0.001**	34.2%	36.0%	36.2%	33.2%	<0.001
Adaptation to new learning experience	48.2%	50.2%	45.2%	37.0%	**<0.001**	51.2%	49.3%	50.8%	45.7%	**<0.001**

**Academic work**										
Timely response of teaching staff	46.1%	48.0%	43.1%	34.4%	**<0.001**	47.4%	46.6%	49.5%	43.4%	**<0.001**
Extent of study workload	48.3%	49.3%	48.2%	43.4%	**<0.001**	46.3%	48.3%	51.6%	48.5%	**<0.001**
Satisfaction with support of teaching staff	48.4%	50.3%	45.9%	36.2%	**<0.001**	49.9%	48.8%	52.0%	45.9%	**<0.001**
Satisfaction with support of support staff	43.1%	44.9%	40.5%	32.3%	**<0.001**	45.4%	44.2%	45.3%	40.9%	**<0.001**

**Academic life**										
Access to a computer	16.9%	18.4%	10.3%	15.3%	**<0.001**	16.3%	15.5%	18.2%	18.4%	**<0.001**
Access to a good Internet connection	41.8%	43.6%	37.8%	32.8%	**<0.001**	44.9%	41.4%	44.2%	40.9%	**<0.001**
Browsing online information	35.6%	37.4%	30.2%	28.7%	**<0.001**	37.8%	35.6%	36.8%	35.0%	0.013
Using online teaching platforms	43.8%	45.3%	41.6%	36.0%	**<0.001**	48.4%	44.4%	44.8%	42.5%	**<0.001**

**Social life**										
Close family member	45.2%	44.9%	45.6%	56.1%	**<0.001**	47.1%	45.2%	46.0%	45.3%	0.233
Someone I live with (e.g., a roommate)	39.1%	39.1%	39.1%	45.0%	**<0.001**	37.5%	38.6%	39.6%	41.4%	<0.001
Close friend	51.3%	50.4%	54.8%	64.4%	**<0.001**	50.8%	51.2%	52.3%	53.0%	0.070
Social networks	39.9%	38.9%	44.0%	51.4%	**<0.001**	38.6%	41.3%	40.0%	40.0%	0.036
**Emotional life**										
Bored	56.3%	55.0%	64.2%	64.0%	**<0.001**	56.8%	59.1%	55.8%	56.1%	**<0.001**
Anxious	55.7%	54.9%	61.2%	62.5%	**<0.001**	53.0%	57.8%	56.4%	55.9%	**<0.001**
Hopeful	58.6%	58.5%	62.5%	61.9%	**<0.001**	61.8%	61.3%	58.6%	56.9%	**<0.001**
Frustrated	58.6%	57.9%	64.2%	65.7%	**<0.001**	58.2%	61.1%	59.3%	57.7%	**<0.001**

**Personal circumstances**										
Professional career in the future	51.9%	51.4%	56.8%	56.9%	**<0.001**	51.5%	54.3%	51.9%	51.6%	<0.001
Study issues	55.8%	55.1%	60.9%	62.5%	**<0.001**	55.5%	57.8%	56.5%	55.2%	0.004
Personal finances	55.0%	54.4%	60.5%	60.5%	**<0.001**	54.0%	57.1%	55.8%	54.9%	0.005
Future education	55.3%	54.3%	62.3%	61.8%	**<0.001**	54.7%	57.8%	55.8%	54.3%	**<0.001**

**Role of institutions**										
Government	53.8%	53.9%	55.9%	56.7%	0.009	55.7%	54.6%	54.2%	55.1%	0.401
University	53.2%	53.4%	55.6%	54.9%	0.018	55.5%	53.7%	54.2%	53.8%	0.332
Banks	50.9%	50.8%	52.6%	57.5%	**<0.001**	51.8%	52.9%	51.3%	50.2%	0.004
Hospitals	42.6%	42.3%	44.3%	50.3%	**<0.001**	43.1%	43.1%	42.8%	43.9%	0.593

**Measures of institutions**										
Emergency support for vulnerable population	25.4%	25.0%	27.8%	29.1%	**<0.001**	23.1%	25.9%	25.3%	27.1%	<0.001
Childcare for essential workers	28.0%	28.0%	29.1%	30.3%	0.063	27.4%	28.8%	27.7%	28.8%	0.152
Financial assistance for renters	34.2%	33.9%	36.7%	40.1%	**<0.001**	32.2%	35.2%	34.7%	35.0%	0.016
Deferred monthly payments	33.9%	33.4%	37.5%	39.0%	**<0.001**	33.5%	35.1%	33.4%	34.8%	0.034

Note: Bold values denote statistical significance at the 0.05 level (after a Bonferroni p-value correction).

## 2 Experimental Design, Materials and Methods

The online questionnaire was grounded on the European Students’ Union Survey [13] and extended with selected elements that facilitated detailed understanding of additional personal and financial circumstances as well as the perception of support measures and behaviour changes during the Covid-19 pandemic's first wave [1]. The questionnaire was initially designed in English and subsequently, with the help of native speakers who are also fluent in English, translated into six other languages, i.e., Italian, North Macedonian, Portuguese, Romanian, Spanish and Turkish [9].

The survey targeted all higher education students. With the aim of reaching a wide range of students, a non-probabilistic sampling technique, specifically convenience sampling, was utilized [14]. This process was facilitated by promoting the online questionnaire on various university communication systems around the world as well as on social media [1]. Detailed information about the survey was available to the students before they gave their informed consent and thereby confirmed their participation in the survey. The data collection was conducted via the open-source web application 1KA (One Click Survey; www.1ka.si). Carried out between 5 May 2020 and 15 June 2020, the data collection came at a time when most countries were experiencing the arduous lockdown restrictions. The data preparation, aggregation, cleaning process, and calculations were performed in the Python programming language utilizing the Pandas and Numpy libraries [15].

## Ethics Statements

All participants were informed about the survey, including its details, and provided their informed consent before participating. Participants agreed to participate in the survey by clicking on a “next page” button, as was explicitly stated on the initial page of the online questionnaire. Participation in the survey was anonymous and voluntary, implying participants could withdraw from the survey without any consequences. Given the data-protection rules, the survey was available to participants being at least 18 years old and enrolled in a higher education institution. The procedures of this survey comply with the provisions of the Declaration of Helsinki regarding research on human participants. Ethical Committees of several of the higher education institutions involved approved this study, such as the University of Verona (protocol number: 152951), ISPA – Instituto Universitário (Ethical Clearance Number: I/035/05/2020), University of Arkansas (IRB protocol number: 2005267431), Walter Sisulu University (Ethical Clearance Number: REC/ST01/2020) and Fiji National University (CHREC ID: 252.20).

## CRediT authorship contribution statement

**Aleksander Aristovnik:** Conceptualization, Investigation, Writing – review & editing, Supervision, Project administration, Funding acquisition. **Damijana Keržič:** Investigation, Writing – review & editing. **Dejan Ravšelj:** Investigation, Data curation, Writing – original draft, Writing – review & editing, Project administration. **Nina Tomaževič:** Investigation, Writing – review & editing. **Lan Umek:** Methodology, Software, Validation, Formal analysis, Investigation, Resources, Data curation, Writing – review & editing, Visualization.

## Declaration of Competing Interest

The authors declare that they have no known competing financial interests or personal relationships that could have appeared to influence the work reported in this paper.
